# Evaluation of 3-hydroxypropionate biosynthesis in vitro by partial introduction of the 3-hydroxypropionate/4-hydroxybutyrate cycle from *Metallosphaera sedula*

**DOI:** 10.1007/s10295-016-1793-z

**Published:** 2016-06-14

**Authors:** Ziling Ye, Xiaowei Li, Yongbo Cheng, Zhijie Liu, Gaoyi Tan, Fayin Zhu, Shuai Fu, Zixin Deng, Tiangang Liu

**Affiliations:** 1 Key Laboratory of Combinatorial Biosynthesis and Drug Discovery, Ministry of Education, Wuhan University School of Pharmaceutical Sciences, Wuhan, 430071 People’s Republic of China; 2 Hubei Engineering Laboratory for Synthetic Microbiology, Wuhan Institute of Biotechnology, Wuhan, 430075 People’s Republic of China; 3 J1 Biotech, Co., Ltd, Wuhan, 430075 People’s Republic of China; 4 Hubei Provincial Cooperative Innovation Center of Industrial Fermentation, Wuhan, 430068 People’s Republic of China; 5 The State Key Laboratory of Microbial Metabolism, Shanghai Jiao Tong University, Shanghai, 200030 People’s Republic of China

**Keywords:** 3-Hydroxypropionate, 3-Hydroxypropionate/4-hydroxybutyrate cycle, *Escherichia coli*, *Metallosphaera sedula*, Metabolic engineering

## Abstract

The chemical 3-hydroxypropionate (3HP) is an important starting reagent for the commercial synthesis of specialty chemicals. In this study, a part of the 3-hydroxypropionate/4-hydroxybutyrate cycle from *Metallosphaera sedula* was utilized for 3HP production. To study the basic biochemistry of this pathway, an in vitro-reconstituted system was established using acetyl-CoA as the substrate for the kinetic analysis of this system. The results indicated that 3HP formation was sensitive to acetyl-CoA carboxylase and malonyl-CoA reductase, but not malonate semialdehyde reductase. Also, the competition between 3HP formation and fatty acid production was analyzed both in vitro and in vivo. This study has highlighted how metabolic flux is controlled by different catalytic components. We believe that this reconstituted system would be valuable for understanding 3HP biosynthesis pathway and for future engineering studies to enhance 3HP production.

**Electronic supplementary material:**

The online version of this article (doi:10.1007/s10295-016-1793-z) contains supplementary material, which is available to authorized users.

## Introduction

The chemical 3-hydroxypropionic acid (3HP) is an important building block and is ranked among the top third of the 12 platform chemicals selected by the US Department of Energy [42]. The bifunctionality of 3HP makes it a versatile platform chemical for numerous applications, including the production of acrylic acid, acryl amide, malonic acid, and 1,3-propanediol [10]. Also, 3HP is a useful starting material for cyclization and polymerization reactions to produce propiolactone, polyesters, poly(3-hydroxypropionate), and other oligomers [18, 22, 32]. Recently, there has been great interest in producing 3HP at an industrial scale from renewable sources, instead of via traditional chemical synthesis.

Several biosynthetic pathways involved in glycerol, glucose, or carbon dioxide metabolism have been proposed for 3HP production [7, 16, 24]. Although significant advances have been made in manipulating microorganisms to produce useful products from carbon dioxide and hydrogen, this strategy is challenging, and few successful trials have been reported. Until now, exploring non-photosynthetic routes for biological fixation of carbon dioxide into valuable industrial chemical precursors and fuels is moving from concept to reality [15]. Recently, an engineered *Pyrococcus furiosus* strain was shown to be able to use hydrogen gas and incorporate carbon dioxide into 3HP by exploiting microbial hyperthermophilicity [14, 19]. Also, the conversion of glycerol to 3HP via glycerol dehydratase and aldehyde dehydrogenase has been investigated extensively using recombinant *Escherichia coli* strains [34–36]. Through systematic engineering of the glycerol dehydrogenase GabD4 from *Cupriavidus necator*, industrial-scale yields of 3HP from glycerol have been achieved [8]. Additionally, the US-based agricultural company Cargill proposed seven important biochemical pathways for 3HP production from glucose via different intermediates, including lactate, glycerate, propionate, beta-alanine, and malonyl-CoA [31]. However, because the reaction is thermodynamically unfavorable, only a few strains have been constructed that can further process the intermediates lactate, glycerate, or propionate [22]. Recently, a synthetic pathway was engineered and optimized for the de novo biosynthesis of beta-alanine and its subsequent conversion into 3HP using a novel beta-alanine–pyruvate aminotransferase discovered in *Bacillus cereus*. This synthetic pathway was expressed in *Saccharomyces cerevisiae*, enabling the production of 13.7 g/L 3HP [5].

Utilizing the component enzymes of the 3-hydroxypropionate or 3-hydroxypropionate/4-hydroxybutyrate cycles to reduce the common intracellular intermediate malonyl-CoA is another attractive route for biosynthetic 3HP production. The metabolite 3HP is a key intermediate in the 3-hydroxypropionate and 3-hydroxypropionate/4-hydroxybutyrate cycles, which are two of the six pathways responsible for autotrophic carbon dioxide fixation [11, 37]. The 3-hydroxypropionate cycle was first observed in the thermophilic, phototrophic eubacterium *Chloroflexus aurantiacus*, which secretes 3HP during phototrophic growth [17]. In this cycle, carbon dioxide is fixed by acetyl-CoA carboxylase (Acc) and propionyl-CoA carboxylase. The newly formed malonyl-CoA metabolite is reduced to 3HP via a bifunctional enzyme, with both alcohol and aldehyde dehydrogenase activities [12]. In 2007, a fifth autotrophic fixation pathway for carbon dioxide, the 3-hydroxypropionate/4-hydroxybutyrate cycle, was discovered in *Metallosphaera sedula* [4]. In *M. sedula*, malonyl-CoA is reduced to 3HP via two separate enzymes, namely malonyl/succinyl-CoA reductase (Mcr) and malonate semialdehyde reductase (Msr). Utilizing the malonyl-CoA pathway to produce 3HP is expected to have some advantages. For example, this pathway is easy to implement, as only one or two enzymatic steps are used to reduce the malonyl-CoA intermediate. Additionally, various C_5_ and C_6_ sugars derived from lignocellulosic biomass can be used as raw materials for 3HP production, as acetyl-CoA is a common intermediate in sugar metabolism. Furthermore, a high recovery of carbon resource is expected from glucose-based pathway, because the carbon dioxide released during glycolysis is reabsorbed in the acetyl-CoA carboxylase-mediated reaction.

In metabolic engineering, discovering and targeting kinetic bottlenecks and stoichiometric inefficiencies are very important. However, the methods used to accomplish these goals are sometimes labor-intensive combinations of molecular cloning and high-throughput screening. To simplify this process, a cell-free system was developed. This system has been applied to transient or steady-state analysis and manipulation of substrates, cofactors, allosteric regulators, and enzyme levels in fatty acid biosynthesis, and these analyses could be completed over a span of a few hours [30]. Subsequently, an escalated in vitro-reconstituted system was developed for the precise analysis of different elements related to fatty acid synthesis [43, 48]. This in vitro-reconstituted system could provide data on critical parameters easily and rapidly, thereby becoming an ideal approach for identifying the rate-limiting step of an optimized system and for understanding any biosynthetic pathway at the biochemical level. This strategy was also extended to cyanobacteria for the overproduction of fatty acids [23] and to *E. coli* for the overproduction of alkenes and alkanes through the iterative polyketide pathway [29].

**Fig. 1:**
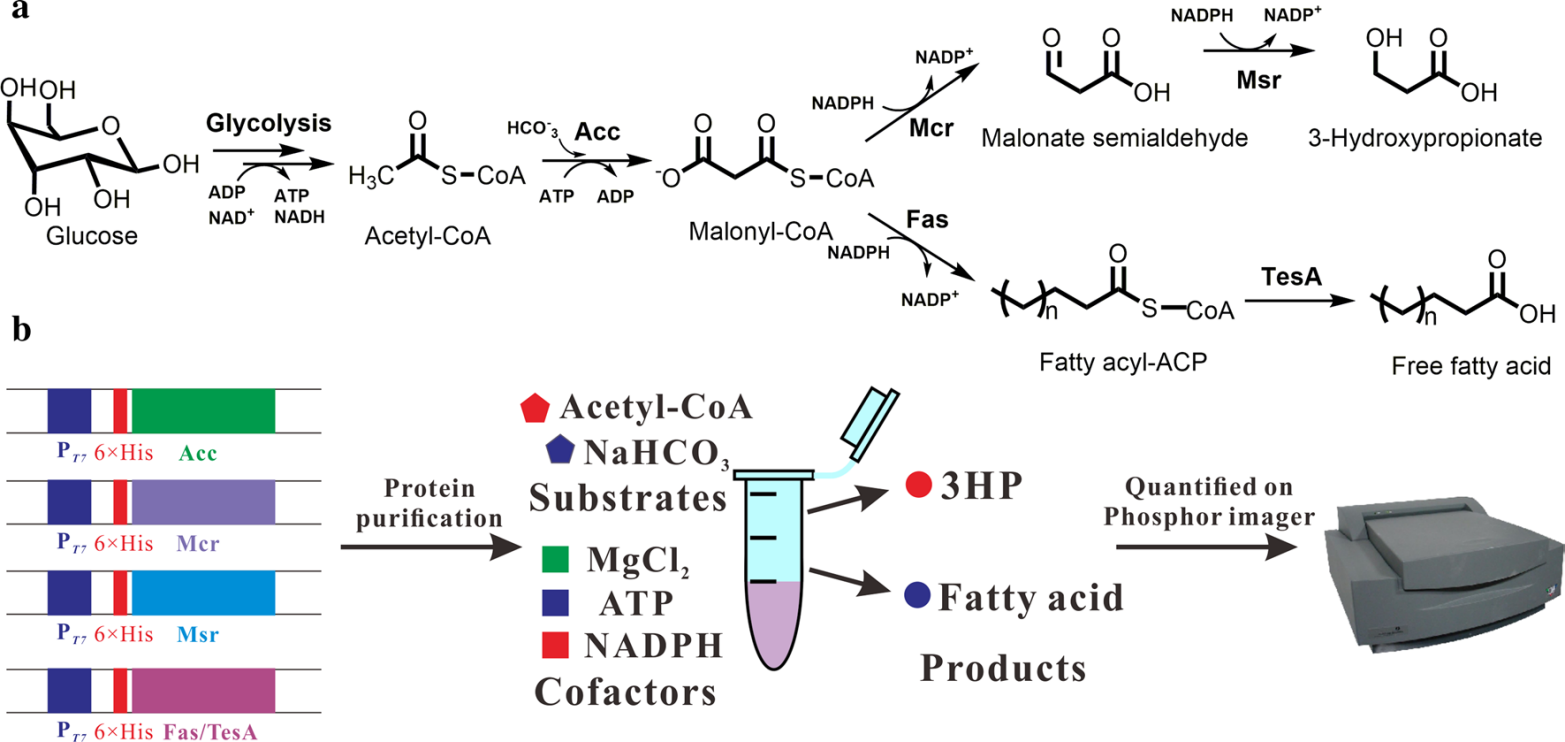
3-Hydroxypropionate (3HP) biosynthesis pathway from glucose through malonyl-CoA. **a** The in vivo 3HP biosynthesis pathway. **b** The in vitro reconstitution assay. *Acc* acetyl-CoA carboxylase, *Mcr* malonyl-CoA reductase from *Metallosphaera sedula*; *Msr* malonate semialdehyde reductase, *Fas* fatty acid synthetase, *TesA* thioesterases

In this study, a part of the 3-hydroxypropionate/4-hydroxybutyrate cycle from *M. sedula* was utilized for 3HP production which allowed for adjusting the ratio between MCR and MSR to obtain the best catalyzing results (Fig. 1). An in vitro-reconstituted system for 3HP biosynthesis was established to assess this pathway for a better understanding of this system. We also analyzed the competition between 3HP formation and fatty acid production based on this in vitro-reconstituted system. Finally, we blocked fatty acid synthesis in vivo to enhance 3HP production.

## Materials and methods

### Materials

DNA polymerase and restriction endonucleases were purchased from New England BioLabs (Ipswich, MA). T4 DNA ligase was purchased from Fermentas (Pittsburgh, PA, USA). QIAprep Spin Miniprep Kits, QIAquick PCR Purification Kits, and gel-extraction kits were obtained from Qiagen (Hilden, Germany). The 3HP standard was purchased from TCI, Inc. (Tokyo, Japan). All other reagents used in the in vitro experiments were purchased from Sigma-Aldrich. [1-^14^C]acetyl-CoA (55 mCi/mmol) was obtained from American Radiolabeled Chemicals, Inc. (St. Louis, MO). Genomic DNA of *M. sedula* strain DSM 5348 was purchased from the German Collection of Microorganisms and Cell Cultures (DSMZ, Braunschweig, Germany).

### Plasmid construction

The plasmids used in this study are listed in Table 1, and the sequences of all oligonucleotides are shown in Table 2. The gene *Msed_0709* (GenBank gene ID: 5103747), which encodes Mcr, was codon-optimized (GenBank accession number: KT962989) and synthesized by GENEWIZ Corp (Suzhou, China). Then, *Msed_0709* was amplified by polymerase chain reaction (PCR) using the forward primer *Nde*I-Mcr-F and reverse primer *Eco*RI-*Spe*I-Mcr-R. *Msed_1993* (GenBank gene ID: 5103380), which encodes Msr, was amplified from *M. sedula* genomic DNA [3] using the forward primer *Nde*I-Msr-F and reverse primer *Hin*dIII-Msr-R. The *Nde*I/*Eco*RI-digested *mcr* gene was inserted into the pET28 vector (Novagen) to generate plasmid pZL37, which was then used to overexpress the Mcr protein as an N-terminal 6 × His-tagged fusion protein. The *Nde*I/*Hin*dIII-digested *msr* gene was inserted into pET28 to generate plasmid pYW4, which was then used to overexpress the Msr protein as an N-terminal 6 × His-tagged fusion protein. Then, the *Xba*I/*Hin*dIII-digested *msr* gene from pYW4 was subcloned into *Spe*I/*Hin*dIII-digested pZL37, yielding the plasmid pZL38, which was then used to overexpress both the Mcr and Msr proteins simultaneously. The pBR322 origin of replication in pET21a was replaced with the p15A origin using the following method. The pET21a backbone fragment was amplified using the forward primer pET21a-F and reverse primer pET21a-R, while the p15A origin-of-replication fragment was amplified using the forward primer p15A-F and reverse primer p15A-R. Subsequently, these two fragments were assembled using a simple cloning method described previously [47] to yield plasmid pXL010. The ketoacyl-ACP synthase gene *fabF* (GenBank gene ID: 946665) was amplified using the forward primer *Bam*HI-FabF-F and reverse primer *Xho*I-FabF-R from *E. coli* genomic DNA. The ketoacyl-ACP synthase gene *fabH* (GenBank gene ID: 946003) was amplified using the forward primer *Nde*I-FabH-F and reverse primer *Bam*HI-FabH-R from *E. coli* genomic DNA. Then, the *Bam*HI/*Xho*I-digested *fabF* gene and the *Nde*I/*Bam*HI-digested *fabH* gene were separately inserted into pXL010 to yield plasmids pXL035 and pXL036. The detailed construction diagram is listed in Fig. S1.

**Table 1:** Plasmids and strains used in this study

Name	Genotype/properties	Resource
pET28a(+)	pBR322 origin, Kan^R^, P_*T7*_	Novagen
pXL010	pET21a(+); replace the pBR322 origin with p15A origin	This study
pZL37	pET28a; P_*T7*_: *N*-terminal his6-tag *mcr*	This study
pYW4	pET28a; P_*T7*_: *N*-terminal his6-tag *msr*	This study
pZL38	pET28a; P_*T7*_: *mcr*–*msr*	This study
pXL035	PXL010; P_*T7*_: *fabF*	This study
pXL036	PXL010; P_*T7*_: *fabH*	This study
BL21 (DE3)	*E. coli* *B dcm ompT hsdS*(*r*_*B*_^−^ *m*_*B*_^−^) *gal*	Invitrogen
MG1655 (DE3)	*E. coli F* ^−^ *λ* ^−^ *ilvG rfb*-*50 rph*-*1*	Invitrogen
XL011	MG1655 (DE3) derivative; {pZL38: P_*T7*_-*mcr*-*msr*}	This study
XL030	MG1655 (DE3) derivative; {pZL38: P_*T7*_-*mcr-msr*; pXL035: P_*T7*_-*fabF*}	This study
XL031	MG1655 (DE3) derivative; {pZL38: P_*T7*_-*mcr*-*msr*; pXL035: P_*T7*_-*fabH*}	This study

**Table 2:** Oligonucleotide primers

Primer name	Sequence (5′–3′)^a^
*Nde*I-Mcr-F	GGACATATGCGCCGTACCCTGAA
*Eco*RI-*Spe*I-Mcr-R	CTTGAATTCACTAGTTTAGCGTTTATCAATATAGC
*Eco*RI-*Spe*I-F	CGCCATATGACTGAAAAGGTATCTGT
*Hin*dIII-Msr-R	CCCAAGCTTTTATTTTTCCCAAACTAGTT
pET21a-F	ATCTTCCAGGAAATCTCCGCCCCGGATATCAACGCCAGCAACGCGGCCTTTT
pET21a-R	TCATCTTATTAATCAGATAAAATATTTGATATCGAAGATCCTTTGATCTTTTCTACGG
p15A ori-F	CCGTAGAAAAGATCAAAGGATCTTCGATATCAAATATTTTATCTGATTAATAAGATGA
p15A ori-R	AAAAGGCCGCGTTGCTGGCGTTGATATCCGGGGCGGAGATTTCCTGGAAGAT
*Bam*HI-FabF-F	TATACGGATCCATGTCTAAGCGTCGTGTAGTTG
*Xho*I-FabF-R	CGAGCCTCGAGTTAGATCTTTTTAAAGATCAAAGAAC
*Nde*I-FabH-F	GACGACATATGTATACGAAGATTATTGGTACTG
*Bam*HI-FabH-R	ATATAGGATCCCTAGAAACGAACCAGCGCGG

^a^For plasmid construction via restriction enzyme digestion and ligation, complementary sequences were designed using the Primer Premier 5 software, and suitable restriction sites and protective bases were introduced; for plasmid construction via the simple cloning method, complementary sequences were designed using the Primer Premier 5 software, flanked by the homologous sequence. The restriction sites and homologous sequence used for cloning are underlined

### Strains and media

The strains constructed and used in this study are listed in Table 1. *E. coli* MG1655 (DE3) was used as the background strain for 3HP production. *E. coli* XL1-Blue was used to propagate the recombinant plasmids.

*Escherichia coli* transformants were selected in LB medium (5 g/L yeast extract, 10 g/L tryptone, and 10 g/L NaCl) with the appropriate antibiotics (50 μg/mL kanamycin and/or 100 μg/mL ampicillin). Modified M9 medium was used for shake-flask cultivations and was prepared (per liter) with 6 g-Na_2_HPO_4_, 3-g KH_2_PO_4_, 0.5-g NaCl, 1-g NH_4_Cl, 1-mM MgSO_4_, 10-mg vitamin B1, 0.1-mM CaCl_2_, 5-g yeast extract, 20 g glucose, and 1 mL of 1000 × Trace Metal Mix (27-g FeCl_3_·6H_2_O, 2-g ZnCl_2_·4H_2_O, 2-g CaCl_2_·2H_2_O, 2-g Na_2_MoO_4_·2H_2_O, 1.9-g CuSO_4_·5H_2_O, 0.5-g H_3_BO_3_, and 100-mL HCl per liter water), as described previously [2]. The initial pH was adjusted to 7.0 with 5 M NaOH.

### Protein purification

To purify the Mcr and Msr proteins, *E. coli* BL21 (DE3) was transformed with plasmids pZL37 and pYW4. The transformed cells were grown in LB media containing 50 μg/mL kanamycin at 37 °C until the OD_600_ reached approximately 0.6. Then, the cells were allowed to cool to 18 °C and induced with 0.1-mM isopropyl-β-d-thiogalactopyranoside (IPTG) for 16–18 h at 18 °C. The cells were centrifuged (6000 rpm, 10 min, 4 °C), resuspended in 35 mL of Buffer A (50 mM Tris, 300 mM NaCl, 4 mM β-mercaptoethanol, pH 8.0), and lysed by sonication. Cellular debris was removed by centrifugation (19,000 rpm, 60 min, 4 °C). The supernatant was loaded into a 5-mL gravity flow column, and the proteins were sequentially eluted with 20 mL of Buffer A (50 mM Tris, 300 mM NaCl, 4 mM β-mercaptoethanol, pH 8.0) supplemented with 50, 150, 300, and 500 mM imidazole. Purified proteins were buffer-exchanged with storage buffer (100 mM phosphate, 10 % glycerol, pH 7.6) and concentrated by centrifugation using an Amicon^®^ Ultra-4 filter (10 kDa, GE Healthcare). Freshly purified proteins were frozen and stored at −80 °C. The four proteins (AccA/B/C/D) expressed in *E. coli* cells were purified as described previously [26]. The ten individual proteins of *E. coli* fatty acid synthetase (Fas) were purified as described previously [48]. Protein concentrations were measured with a Pierce™ BCA Protein Assay Kit (Thermo Scientific) using bovine serum albumin (BSA) to generate a standard curve.

### In vitro reconstitution of the 3HP-synthesis system

In vitro assays with the reconstituted system were performed as described previously [29, 48]. Briefly, the reactions were performed in 50-mM phosphate buffer (pH 7.6) in a volume of 140 μL, with 5-mM NADPH, 1-mM TCEP, 5-mM ATP, 10-mM NaHCO_3_, 10-mM MgCl_2_, 1-mM acetyl-CoA (with 5 % [1-^14^C] acetyl-CoA), 3 or 10 μM each of AccA/B/C/D, and increasing concentrations of Mcr and/or Msr for the titration assays. At various intervals, 20 μL of the reaction mixture was withdrawn and quenched with 180 μL of an 80:80:20 (v/v) solution of methanol:isopropanol:acetic acid. The samples were dried and resuspended in 30 μL of methanol, and then spotted on a silica-gel thin-later chromatography plate. Finally, the samples were chromatographed using a solvent system comprised of a 70:40:2 (v/v) mixture of chloroform:methanol:acetic acid. Radioactivity was quantified on a Packard Phosphor imager (GE Healthcare), using [1-^14^C]acetyl-CoA as the calibration standard.

For competitive radioassays between 3HP and fatty acid production, the reaction mixtures included 3 or 10 μM each of AccA/B/C/D, 0.2 or 1 μM Mcr, 1 μM Msr, 1 μM each of FabA/B/D/F/G/H/I/Z, 10 μM holo-ACP, 10 μM TesA, and/or 200 μM cerulenin. To separate the products (3HP and fatty acid), the samples were first chromatographed using a mobile phase comprised of a 50:60:2 (v/v) mixture of hexane:diethylether:acetic acid, followed by a second mobile phase comprised of a 70:40:2 (v/v) mixture of chloroform:methanol:acetic acid.

### Flask fermentation procedure

The *E. coli* strain was cultivated in modified M9 minimal media (described above) for flask fermentation. Single colonies were inoculated in 5 mL of LB media and cultured overnight at 37 °C. Then, 1 mL of seed culture was added to 100 mL of M9 media containing the appropriate antibiotics (50 μg/mL kanamycin and/or 100 μg/mL ampicillin) in a 500-mL flask and grown at 37 °C and 220 rpm. IPTG (0.1 mM) was added when the OD_600_ reached 0.7. Cerulenin (10 mg/L) was added at 4 h after induction. All experiments were performed in triplicate.

### Analysis of 3HP production by HPLC

The 3HP concentration was analyzed using a previously described method [5]. The sample was separated using a Dionex UltiMate 3000 HPLC system (Thermo Scientific, USA) and analyzed for 35 min using an Aminex HPX-87H ion-exclusion column (Bio-Rad, Hercules, USA) with a mobile phase of 5-mM H_2_SO_4_ and a flow rate of 0.5 mL/min. The temperature of the column was maintained at 50 °C. The refractive index at 45 °C and the UV absorption (RS Variable Wavelength Detector, UltiMate 3000, Thermo Scientific, USA) at 210 nm were measured. The concentrations of 3HP were detected using a refractive index detector (Shodex RI-101, Japan) and were verified by measuring their UV spectra in comparison with the spectrum of the 3HP standard.

## Results

### Effects of Mcr and Msr concentrations on the 3HP-synthesis rate

The metabolite 3HP was previously described as a key intermediate of the 3-hydroxypropionate/4-hydroxybutyrate cycle [4]. However, this separated system from *M. sedula* has not been investigated to produce 3HP. To obtain detailed biochemical information regarding the capacity of this cycle for 3HP biosynthesis, we generated an in vitro-reconstituted system. Six individual proteins, namely, AccA/B/C/D, Mcr, and Msr, were purified to assemble the 3HP synthetic pathway in vitro. [1-^14^C]acetyl-CoA was used as the substrate.

**Fig. 2:**
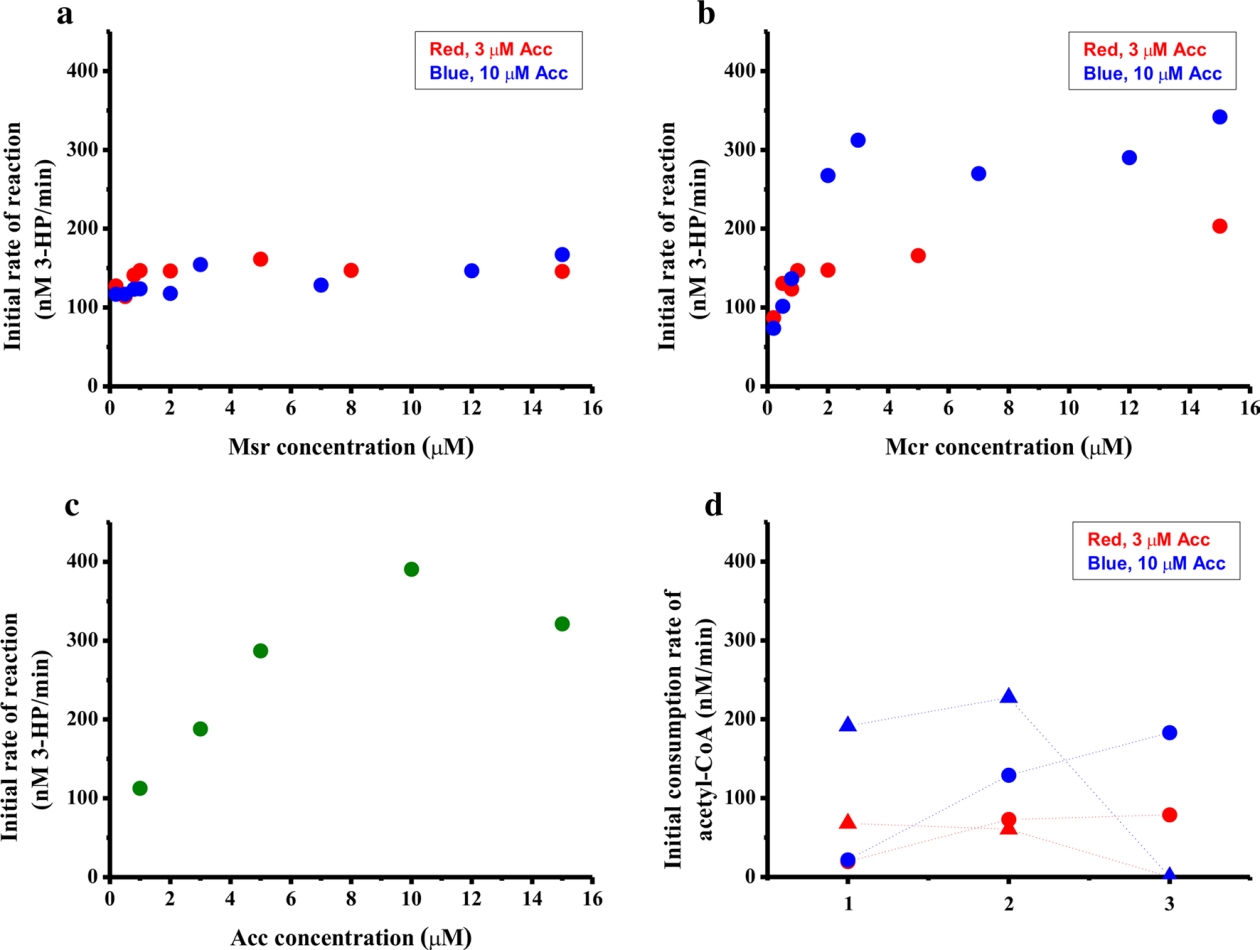
In vitro reconstitution of 3HP production from acetyl-CoA. **a** Titration of Msr; the assay mixture included 1-μM Mcr and 10-μM Acc (*blue*) or 3-μM Acc (*red*). **b** Titration of Mcr; the assay mixture included 1-μM Msr and 10-μM Acc (*blue*) or 3-μM Acc (*red*). **c** Titration of Acc; the assay mixture included 5-μM Mcr and 1-μM Msr. **d** Competitive radioassay between 3HP and fatty acid production. Each assay mixture included 1 μM of each Fab, 10-μM holo-Acp, 10-μM TesA, 1-μM Msr, 10-μM Acc (*blue*) or 3-μM Acc (*red*), and (*1*) 0.2-μM Mcr or (*2*) 1-μM Mcr or (*3*) 1-μM Mcr, and 200-μM cerulenin. *Solid circle*, 3HP; *Solid triangle*, fatty acid

To examine the effects of the Mcr and Msr protein concentrations on the steady-state activity of this reconstituted system, the concentration of Acc was fixed at 10 μM. We first investigated the effect of the 3HP-biosynthesis rate on the concentration of Msr, using a fixed Mcr concentration of 1 μM. The results showed that the level of 3HP increased only slightly when the concentration of Msr was increased from 0.2 to 15 μM (Fig. 2a). We further investigated the dependence of Mcr on 3HP biosynthesis when the concentration of Msr was fixed at 1 μM. The results showed that Mcr had a marked effect when the concentration was varied from 0.2 to 3 μM, resulting in a 4.2-fold increase in the rate of 3HP formation. Mcr concentrations ranging from 3 to 15 μM did not appreciably influence the rate of 3HP synthesis (Fig. 2b). Also, similar results were observed when the Acc concentration was reduced to 3 μM (Fig. 2a, b). These data showed that the reaction catalyzed by Mcr, rather than that catalyzed by Msr, is the critical limiting step in the 3HP biosynthesis pathway.

### Effects of Acc concentration on the 3HP synthesis rate

Conversion of acetyl-CoA to malonyl-CoA by Acc is the initial step in 3HP biosynthesis (Fig. 1). Many researchers have overexpressed this enzyme to increase the production level of downstream products [9, 30, 38, 41]. Therefore, we employed this enzyme in our in vitro-reconstituted system to evaluate the contribution of Acc to 3HP biosynthesis. To prevent limitations caused by downstream proteins, the concentration of Mcr was fixed at 5 μM, while that of Msr was fixed at 1 μM. Acc supplementation increased the 3HP synthesis rate at concentrations below 10 μM, while the 3HP synthesis rate was reduced at Acc concentrations exceeding 10 μM (Fig. 2c). This phenomenon indicated that, although the production of 3HP was dependent on the concentration of Acc, excessive Acc would inhibit 3HP formation.

### Analysis of the competition between 3HP and fatty acid synthesis

Acetyl-CoA is a critical intermediate metabolite in the metabolic network of the cell. Acetyl-CoA is the substrate for the tri-carboxylic acid cycle and a precursor metabolite for amino acid, nucleotide base, and porphyrin synthesis, as well as the substrate for protein acetylation [21]. However, when malonyl-CoA is formed through the condensation of bicarbonate with acetyl-CoA, the product primarily flows into fatty acid synthesis [39]. Thus, fatty acid biosynthesis is the main competitive pathway for exogenously introduced 3HP formation, with malonyl-CoA as the substrate (Fig. 1). In the in vitro titration assays, reconstitution of the fatty acid synthase was performed as described previously [48]. The concentrations of FabA/B/D/F/G/H/I/Z were 1 μM each, and those of holo-ACP and TesA were 10 μM [48].

Here, we first introduced the fatty acid biosynthesis system into the 3HP-formation system and analyzed the competitive relation between 3HP and the fatty acid biosynthesis pathway in vitro. When 0.2-μM Mcr and 1-μM Msr were titrated with the fatty acid synthesis system, an 8.9-fold higher initial consumption rate for fatty acids versus 3HP was observed (Fig. 2d). When 1-μM Mcr and 1-μM Msr were titrated with the fatty acid synthesis system, the ratio was reduced to 1.8 (Fig. 2d). This result indicated that when the Mcr concentration was low, the fatty acid pathway was the main competitive pathway, and when Mcr concentration increased to an equal amount with Msr, the ability of 3HP production was comparable to fatty acid biosynthesis. Also, 200-μM cerulenin, a specific inhibitor of the *fabB*–*fabF* gene products [27], was added to block the production of fatty acids. In this experiment, no fatty acids were produced, and the rate of 3HP production increased marginally (Fig. 2d).

**Fig. 3:**
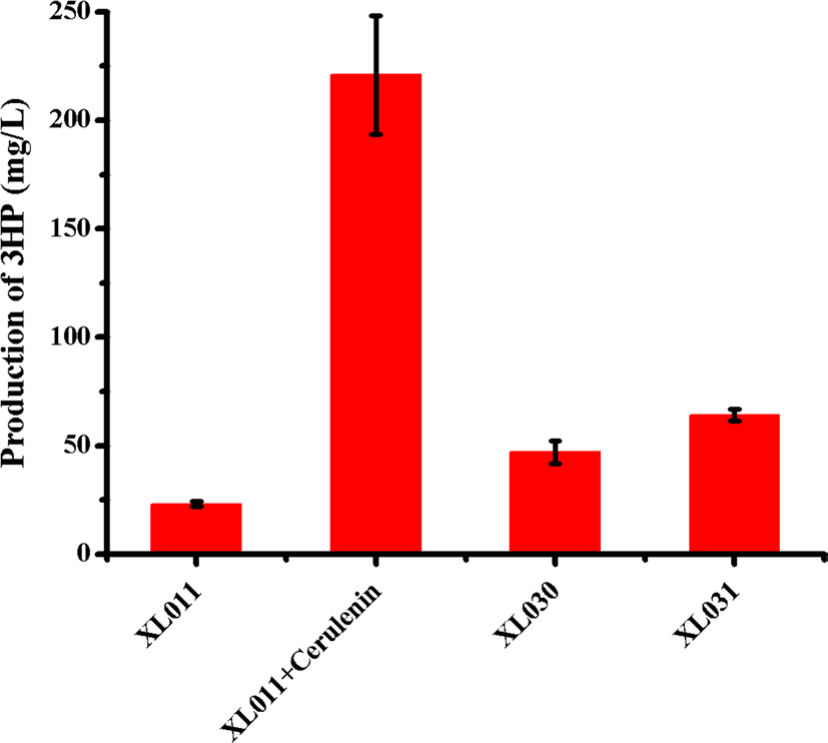
Production of 3HP by blocking fatty acid biosynthesis

To investigate the effect of repression of fatty acid synthases on 3HP biosynthesis in vivo, the production of 3HP was evaluated by repressing FabB and FabF activities using 10 mg/L cerulenin. The 3HP production was identified by NMR (Fig. S2) and quantified via HPLC (Fig. S3). The amount of 3HP produced was approximately 221 mg/L, which is 10-fold that produced by strain XL011 without cerulenin addition (Fig. 3). However, the addition of cerulenin is cost-prohibitive for an industrial scale fermentation process. Therefore, an alternative metabolic engineering strategy was attempted. Previous studies have shown that increasing the amount of FabF or FabH could significantly inhibit the production of fatty acids [48]. When *fabF* was overexpressed, strain XL030 produced 47.0 mg/L 3HP, while a *fabH*-overexpressing strain XL031 produced 64.2 mg/L 3HP (Fig. 3).

## Discussion

In this study, we reconstituted an in vitro synthetic pathway for 3HP production from three purified components using acetyl-CoA as the substrate. Our results indicate that 3HP synthesis is sensitive to Acc and Mcr, but not to Msr. It was reported that the *K*_m_ value and specific activity of Mcr equaled 100 μM and 4.6 μM min^−1^ mg^−1^, respectively [1], while the *K*_m_ value and specific activity of Msr equaled 70 ± 10 μM and 200 μM min^−1^ mg^−1^, respectively [20]. Although lacking the biochemistry parameter of Acc, it has been shown to have a limited specific activity in *E. coli* cell-free extract [9, 30]. All these data are consistent with the results of our in vitro reconstitution experiment. This finding has provided key clues for conducting targeted engineering of 3HP production in future studies. For example, our results suggest that more attention should be paid to the expression levels of Acc and Mcr. Also, we extended the application of the in vitro-reconstituted system to analyze the competition between 3HP formation and fatty acid synthesis. The results revealed that when the Mcr concentration in the system is low, more carbon flows into the fatty acid synthesis system than into the 3HP synthesis pathway. However, when the amount of Mcr is equal to the molar ratio of fatty acid synthase, the 3HP formation rate is similar to that of fatty acid synthesis. This result also implied that the cells were as capable of synthesizing 3HP as they were of synthesizing fatty acids.

Acetyl-CoA carboxylase is a widely used target in different organisms to overproduce various downstream products for metabolic engineering [9, 30, 38, 41]. However, altering the rate-limiting step to improve production is not simply a matter of “the more the better.” The balance of protein expression levels is also essential. It was reported that the expression of the ACA module by low-copy-number plasmids led to higher fatty acid production than with expression via high-copy-number plasmids [44]. In our in vitro titration assays, we found that supplementation of Acc increased the initial rate of 3HP synthesis only within an appropriate range, beyond which 3HP synthesis was inhibited. So, the expression level of Acc should be precisely regulated. In fact, fine-tuning the expression of Acc can be achieved via altering the promoters. Also, development of malonyl-CoA-responsive sensors that control Acc expression levels based on intracellular malonyl-CoA concentrations [28, 45] may hold great promise in overcoming critical limitations of Acc and optimizing 3HP titers and yields.

Blocking fatty acid biosynthesis has been applied previously to increase the production of resveratrol or plant flavonoid polyphenols [25, 27]. In this study, we found that carbon was mainly fluxed into the fatty acid biosynthetic pathway when a low concentration of Mcr was titrated into the fatty acid system. However, the highest 3HP-production rate was achieved only when cerulenin was added. Overexpression of *fabF* or *fabH* yielded only modest improvement. Recently, antisense RNA methods with high interference efficiencies of up to 80 % were developed to enhance the rate of biosynthesis of natural products [46]. Additionally, an RNA-based method, CRISPRi (clustered regularly interspaced palindromic repeats interference), that can efficiently silence a target gene with up to 99.9 % repression in *E. coli* has been developed recently [33]. Application of antisense RNA or CRISPRi technology may represent a better metabolic engineering strategy for increasing 3HP production by blocking fatty acid biosynthesis.

In this work, critical information regarding 3HP formation from acetyl-CoA was obtained using an in vitro-reconstituted system. The competition analysis between fatty acid biosynthesis and 3HP formation indicated that the potential of 3HP production was comparable to that of fatty acid biosynthesis. This study highlights the utility of the in vitro-reconstituted system. More efforts need to be directed at enhancing 3HP production in future work through in vivo metabolic engineering. Some feasible and effective strategies have been exploited in engineering the 3HP pathway in previous studies. Deletion of acetyl-CoA synthetase I and acetyl-CoA synthetase II, which competes with the heterologous pathway for acetyl-CoA, markedly improved 3HP production [40]. The 3HP level was also enhanced in engineered *Saccharomyces cerevisiae* by increasing the availability of the precursor malonyl-CoA and by coupling the production with increased NADPH supply [6]. Besides, combining different metabolic pathways to enhance 3HP production is an attractive strategy. A recombinant *E. coli* has been successfully constructed harboring a 3HP-synthetic pathway that transfers glucose to 3HP via a glycerol intermediate [32]. Engineering 4HB to acetyl-CoA [13] and its subsequent conversion to 3HP might also represent an effective production strategy. Also, exploiting the hyperthermophilicity of *M. sedula* to enhance 3HP production is attractive, and this strategy has been established successfully in *P. furiosus* [19]. These targeted metabolic engineering strategies have demonstrated enhanced 3HP production and have paved the way for future work. In conclusion, combination of the in vitro assay with an in vivo metabolic engineering strategy would further improve 3HP formation.

## Electronic supplementary material

Below is the link to the electronic supplementary material.

Supplementary material 1 (DOCX 2166 kb)

## Abbreviations

3HP: 3-Hydroxypropionate
Acc: Acetyl-CoA carboxylase
Mcr: Malonyl-CoA reductase
Msr: Malonate semialdehyde reductase
Fas: Fatty acid synthetase
TesA: Thioesterase
FabF: Ketoacyl-ACP synthase
FabH: Ketoacyl-ACP synthase
