# Supplementary material for: Evaluation of 3-hydroxypropionate biosynthesis in vitro by partial introduction of the 3-hydroxypropionate/4-hydroxybutyrate cycle from Metallosphaera sedula
Source: J Ind Microbiol Biotechnol. 2016 Jun 14;43:1313–21. doi: 10.1007/s10295-016-1793-z (PMC4983293; doi:10.1007/s10295-016-1793-z)

**Supplementary Material for** **Journal of Industrial Microbiology & Biotechnology**

**Evaluation of 3-hydroxypropionate biosynthesis *in vitro* by partial introduction of the 3-hydroxypropionate/4-hydroxybutyrate cycle from *Metallosphaera sedula***

**Ziling Ye^a,1^, Xiaowei Li^a,1^, Yongbo Cheng^a^, Zhijie Liu^a^, Gaoyi Tan^a^, Fayin Zhu^a^, Shuai Fu^c^, Zixin Deng^a,b,e^, Tiangang Liu ^a,b,d*^**

^a^Key Laboratory of Combinatorial Biosynthesis and Drug Discovery, Ministry of Education, and Wuhan University School of Pharmaceutical Sciences, Wuhan 430071, P. R. China

^b^Hubei Engineering Laboratory for Synthetic Microbiology, Wuhan Institute of Biotechnology, Wuhan 430075, P. R. China

^c^J1 Biotech, Co., Ltd, 430075, P. R. China

^d^Hubei Provincial Cooperative Innovation Center of Industrial Fermentation, Wuhan 430068, P. R. China

^e^The State Key Laboratory of Microbial Metabolism, Shanghai Jiao Tong University, Shanghai 200030, P. R. China

^1^Ye and Li contributed equally to this work.

*E-mail of corresponding author: liutg@whu.edu.cn, Tel/Fax: +86-27-68755086

**Supplementary Methods**

**NMR spectroscopy**

Nuclear magnetic-resonance spectroscopy (NMR) analysis was used to confirm the production of 3HP. Briefly, 10 mL of culture sample of the XL011 strain was centrifuged for 5 min at 4 °C and 20,000 × g to remove cells; the supernatant was then lyophilized. Then, the 3HP fraction was collected through semi-preparative high-performance liquid chromatography (HPLC) and re-lyophilized. The lyophilized sample was re-dissolved in 500 μL of D_2_O. The sample was then analyzed at room temperature on an Agilent 400 DD2 spectrometer calibrated for 1H-NMR. 1H-1H correlation spectroscopy, 1H-13C heteronuclear single-quantum correlation, and 1H-13C heteronuclear multiple-bond correlation 2D NMR spectra were acquired to assign spectral resonances.

**Figure legends**

Fig. S1 The flow diagram of plasmid construction. (a) Plasmid construction for the expression of *mcr* and/or *msr*. (b) Replacement of the pBR322 origin of replication with the p15A origin in pET21a. (c) Plasmid construction for the expression of *fabF* or *fabH*.


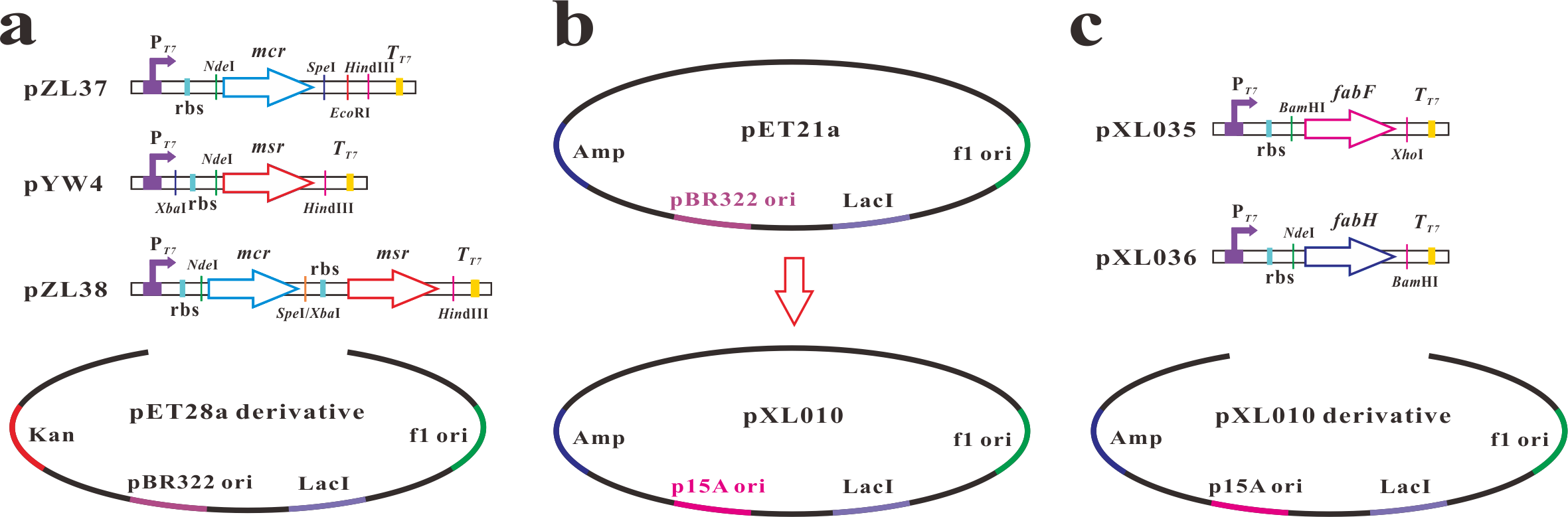


Fig. S2 Identification and confirmation of 3HP produced by *E. coli* in shaking-flask culture by 400-MHz NMR spectrometry. (a) 1H 1D NMR. (b) 1H-1H COSY 2D NMR. (c) 1H-13C HMQC 2D NMR. (d) 1H−13C HMBCAD 2D NMR.


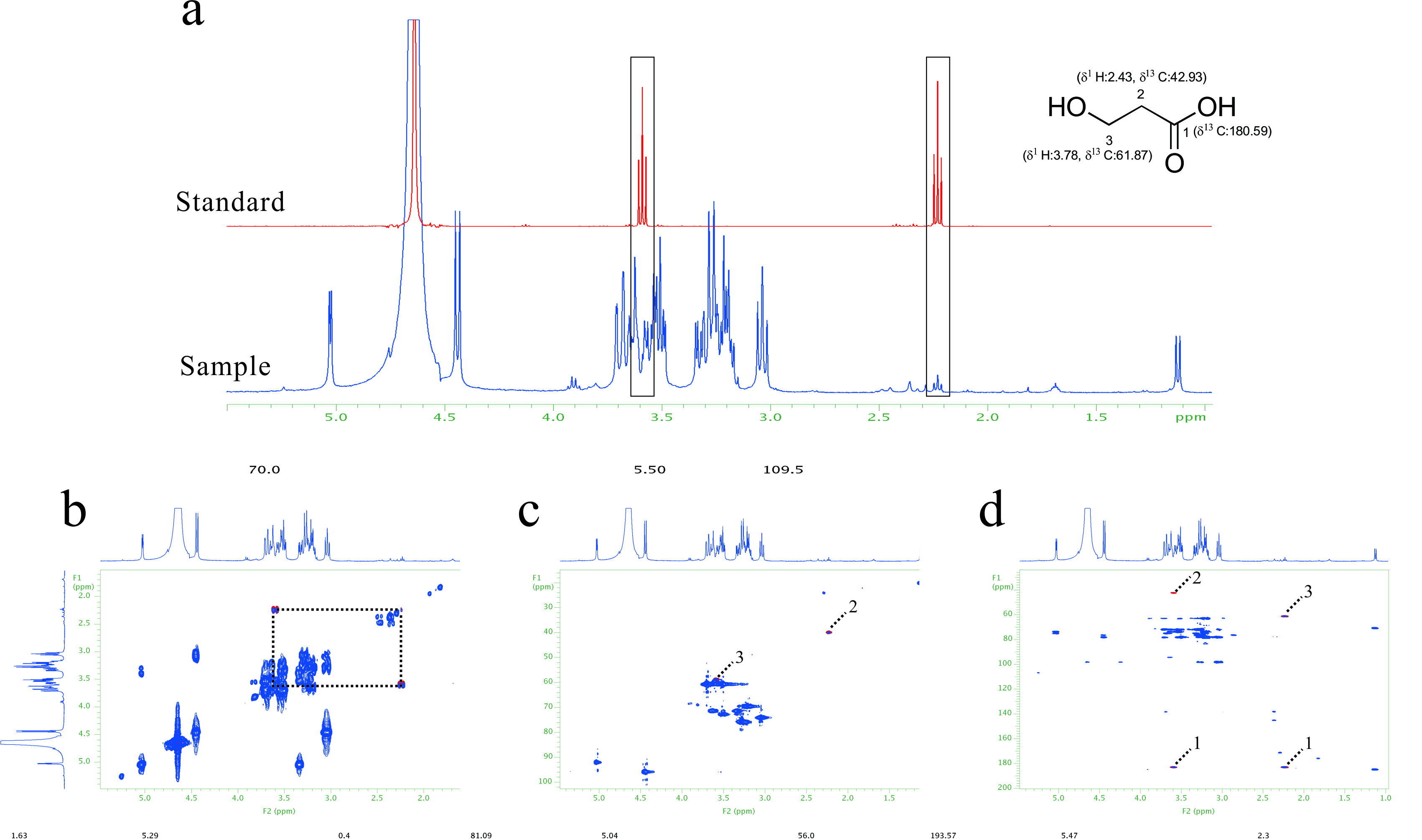


Fig. S3 HPLC chromatograms of flask fermentation. The sample was separated using a Dionex UltiMate 3000 HPLC system (Thermo Scientific, USA) and analyzed for 35 min using an Aminex HPX-87H ion-exclusion column (Bio-Rad, Hercules, USA). The 3HP concentrations were detected using a Refractive Index Detector (Shodex RI-101, Japan). (a) Overexpression of *mcr* and *msr*. (b) Overexpression of *fabF*. (c) Overexpression of *fabH*. (d) Addition of cerulenin. (e) 3HP standard.


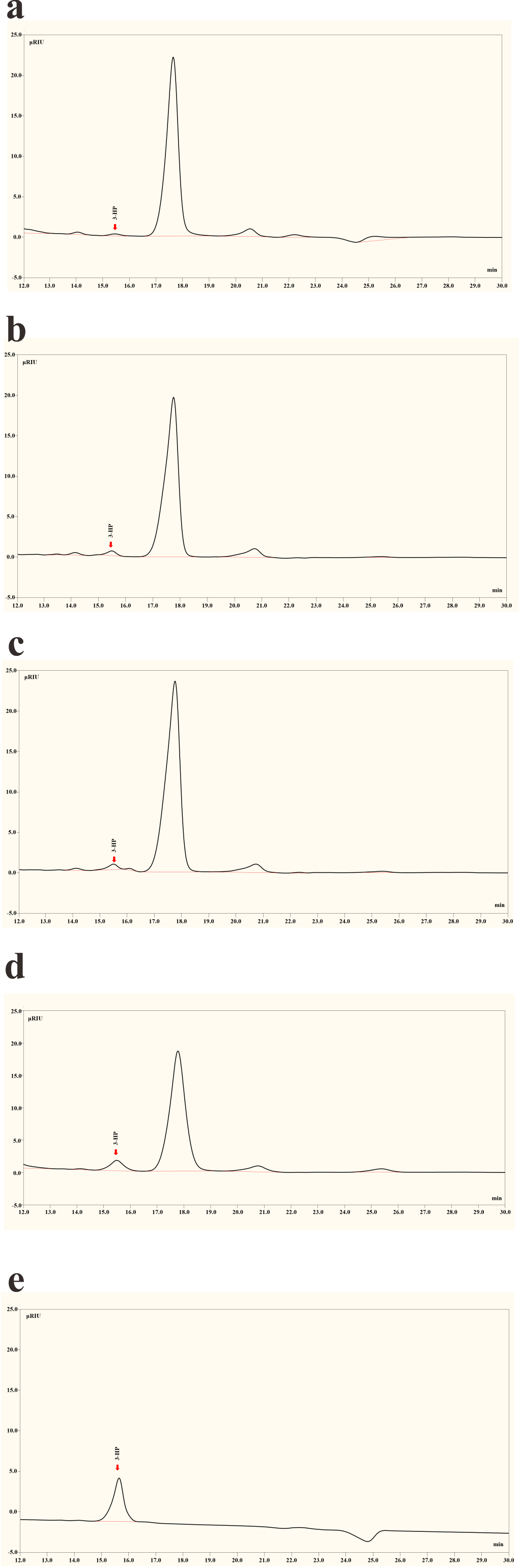

Supplement: Supplementary file 1 — Supplementary material 1 (DOCX 2166 kb) [file 10295_2016_1793_MOESM1_ESM.docx]
